# The Carbon Monoxide Releasing Molecule CORM-2 Attenuates *Pseudomonas aeruginosa* Biofilm Formation

**DOI:** 10.1371/journal.pone.0035499

**Published:** 2012-04-26

**Authors:** Thomas S. Murray, Chinweike Okegbe, Yuan Gao, Barbara I. Kazmierczak, Roberto Motterlini, Lars E. P. Dietrich, Emanuela M. Bruscia

**Affiliations:** 1 Departments of Pediatrics, Yale University School of Medicine New Haven, Connecticut, United States of America; 2 Laboratory Medicine, Yale University School of Medicine New Haven, Connecticut, United States of America; 3 Department of Biological Sciences, Columbia University, New York, New York, United States of America; 4 Internal Medicine, Yale University School of Medicine New Haven, Connecticut, United States of America; 5 INSERM U955, University Paris-Est, Creteil, France; University of Malaya, Malaysia

## Abstract

Chronic infections resulting from biofilm formation are difficult to eradicate with current antimicrobial agents and consequently new therapies are needed. This work demonstrates that the carbon monoxide-releasing molecule CORM-2, previously shown to kill planktonic bacteria, also attenuates surface-associated growth of the Gram-negative pathogen *Pseudomonas aeruginosa* by both preventing biofilm maturation and killing bacteria within the established biofilm. CORM-2 treatment has an additive effect when combined with tobramycin, a drug commonly used to treat *P. aeruginosa* lung infections. CORM-2 inhibited biofilm formation and planktonic growth of the majority of clinical *P. aeruginosa* isolates tested, for both mucoid and non-mucoid strains. While CORM-2 treatment increased the production of reactive oxygen species by *P. aeruginosa* biofilms, this increase did not correlate with bacterial death. These data demonstrate that CO-RMs possess potential novel therapeutic properties against a subset of *P. aeruginosa* biofilm related infections.

## Introduction

Organized bacterial communities or biofilms are formed on both biotic and abiotic surfaces during infections by a wide variety of Gram-positive and Gram-negative pathogens [1]. The bacteria are protected from both the immune system and antimicrobials by an extracellular matrix leading to prolonged colonization of the host [2]. Therefore, new approaches to biofilm eradication and the treatment of chronic biofilm-associated infections are needed. *P. aeruginosa* is an opportunistic Gram-negative pathogen that has served as a model organism to study the dynamics of biofilm assembly and maintenance [3]. Since *P. aeruginosa* causes chronic infection in patients with underlying lung disease such as cystic fibrosis (CF), therapies that disrupt or prevent *P. aeruginosa* biofilm formation are of critical clinical interest [4], [5].

Carbon monoxide (CO) is a product of heme oxygenase activity that serves as an anti-inflammatory signaling molecule in mammalian cells [6], [7] by binding, among others, divalent metals of heme-containing proteins such as guanylate cyclase, mitochondrial cytocrome c oxidase and NADPH oxidase [8], [9], [10]. CO selectively inhibits the expression of pro-inflammatory cytokines, increasing the anti-inflammatory cytokine IL-10 [11], [12], [13] and reducing neutrophil migration in septic lungs by suppressing transendothelial migration [14]. CO inhalation has been reported to exert an anti-inflammatory effect in patients with chronic obstructive pulmonary disease [15]. Exogenous administration of low concentrations of CO (10–500 ppm) by inhalation is currently in a phase I clinical trial to evaluate its potential to reduce acute airway inflammation (NCT00094406).

Due to concerns about delivering gaseous CO, which cannot be precisely controlled and could be toxic to tissue with prolonged exposure, chemical carriers of this gas known as CO-releasing molecules (CO-RMs) have been developed [6], [16]. These carbonyl complexes contain a transition metal bound to CO that is released once in solution. Recent studies in bacteria, including *P. aeruginosa*, *Staphylococcus aureus, and Escherichia coli*, show that CO liberated from CO-RMs binds primarily to the heme moieties of proteins in the electron transport chain resulting in decreased oxygen consumption and rapid cell death [17], [18], [19]. Exposure of *E. coli* to CO-RMs also increases intracellular reactive oxygen species (ROS) resulting in bacterial DNA damage and death [20]. Thus, in addition to their anti-inflammatory properties, CO-RMs potentially represent a novel class of antimicrobials. While the water-soluble CORM-3 efficiently kills planktonic *P. aeruginosa* at concentrations as low as 1 µM [18], the effect of CO-RMs on biofilms, where bacterial metabolism and respiration is likely to be altered, is poorly understood. Biofilm formation by *E. coli* is enhanced after exposure to CO-RMs but whether this is also true for other bacteria is unknown and has implications for developing these drugs as antimicrobials [21]. The purpose of this study was to evaluate the effects of CORM-2, a ruthenium-containing CO-RM soluble in dimethyl sulfoxide (DMSO), on *P. aeruginosa* biofilm formation and surface colonization. Our study demonstrates that CORM-2 attenuates *P. aeruginosa* formation at concentrations that are not toxic for mammalian cells, and that the CORM-2 bactericidal efficiency varies with the *P. aeruginosa* genetic background and growth conditions.

## Results

### CORM-2 kills *P. aeruginosa* PAO1 during planktonic growth and biofilm formation

**Figure 1:**
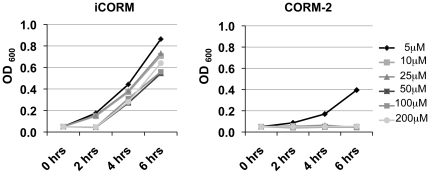
CORM-2 kills planktonic *P. aeruginosa*. The control molecule iCORM has no effect on PAO1 growth in liquid M9 medium with glucose (left panel), while CORM-2 doses >5 µM prevent growth of PAO1 (right panel).

Recent studies have shown that CORM-2, CORM-3 and, to a lesser extent, CORM-371 have previously been shown to kill planktonic *P. aeruginosa* [18], [22]. All these CO-RMs contain a transition metal, either ruthenium (CORM-2 and CORM-3) or manganese (CORM-371). Our data confirm that CORM-2, but not its inactive counterpart iCORM, kills planktonic PAO1 in M9 medium supplemented with glucose. The effect was dose-dependent with a minimal inhibitory concentration of 10 µM (Figure 1). The vehicle alone (DMSO) did not affect growth of PAO1 (data not shown). Colony counts of viable bacteria determined that this killing was rapid with a three-log drop in recovered bacteria at 20 min and no viable bacteria recovered after 30 min (data not shown).

**Figure 2:**
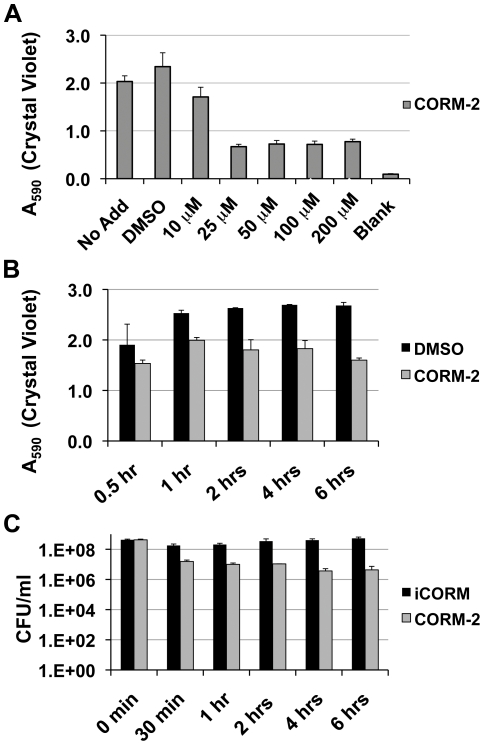
CORM-2 attenuates *P. aeruginosa* biofilm formation. A) Crystal violet (CV) biomass staining decreases after CORM-2 exposure in a dose-dependent manner. Each condition was done in triplicate. B) CV staining of PAO1 biofilms treated with 100 µM CORM-2 demonstrates decreased staining of the biofilm after 2–6 hours of CORM-2 exposure compared with DMSO vehicle where the biofilm increases overtime. C) Colony counts of PAO1 released from the iCORM (100 µM) and CORM-2 (100 µM) treated biofilms demonstrate that there is a sustainable 2-log drop in bacterial viability.

We next assessed whether CORM-2 can impact surface associated *P. aeruginosa*. Crystal violet (CV) staining of PAO1 biofilms formed in plastic wells demonstrated that a 6 h exposure to CORM-2 decreased CV biofilm staining with exposure to concentrations ≥25 µM of CORM-2 showing a statistically significant effect (ANOVA p<0.001) (Figure 2A). Post-test Dunnett's analysis demonstrated that CV staining levels for all samples treated with ≥25 µM CORM-2 were different from the control but not different from each other. The effect of CORM-2 on biofilm formation compared with vehicle was observed as early as 60 min after treatment and was statistically significant at 4 h (p<0.007, paired t-test) (Figure 2B). We next performed colony counts from sonicated biofilms +/− 100 µM iCORM or 100 µM CORM-2 to determine if bacterial cell death contributed to changes in CV of the biofilm. The data demonstrate that there is a statistically significant two-log drop in the number of viable bacteria recovered from the CORM-2 treated biofilm when compared with the initial viable cell counts and 100 µM iCORM (Figure 2C) (ANOVA<0.001, Dunnett's post-test analysis). Cell death is observed as early as 30 min after drug treatment and persists up to 6 h.

### CORM-2 prevents maturation of PAO1 biofilms

**Figure 3:**
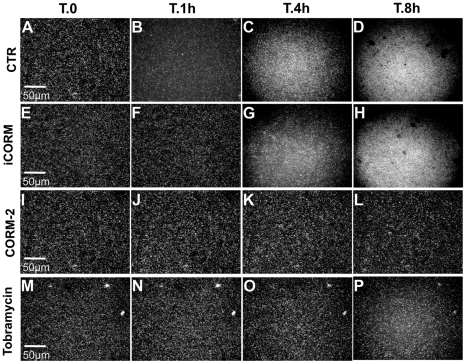
CORM-2 attenuates PAO1 biofilm formation. YFP-*P. aeruginosa* were grown for 16 hours in a glass bottom dish and then visualized at selected locations for 12 additional hours using VivaView. A–D: control (CTR = no addition) biofilm formation; E-H: biofilm formation in presence of iCORM (50 µM); I–L: biofilm formation in presence of CORM2. The CORM-2 (50 µM) treated biofilm remains unchanged compared with the control biofilms that continue to mature; M–P: biofilm formation in presence of tobramycin. T indicates time; h indicates hours. Scale bars = 50 µm.

We next examined the dynamics of biofilm formation after addition of CORM-2, iCORM or tobramycin, a known antibiotic used for *P. aeruginosa* therapy. PAO1-YFP bacteria were incubated in glass bottom dishes for 16 h, then gently washed. Fresh medium with iCORM, DMSO, CORM-2 (50 µM) or tobramycin (100 µg/ml) was added to surface attached bacteria. Fluorescence microscopy was used to image the biofilm at a single location over time. We observed that PAO1 biofilms do not continue to mature in the presence of CORM-2 compared with biofilms formed in absence of the drug (compare Figure 3A–D and 3I–L). CORM-2 is as effective as tobramycin (100 µg/ml) in preventing bacterial growth, thus maturation of PAO1 biofilms (compare Figure 3 I–L to M–P; see also Figure S1).

No effect on biofilm maturation was observed in the presence of the iCORM vehicle control (Figure 3 E–H) or DMSO (data not shown). These data suggest that the release of CO mediated by CORM-2 prevents further biofilm development, similar to the growth inhibition observed during planktonic growth. This observation was confirmed by quantification of bacterial fluorescence (Figure S1).

### CORM-2 attenuates PAO1 microcolony formation on human bronchial epithelial cells

**Figure 4:**
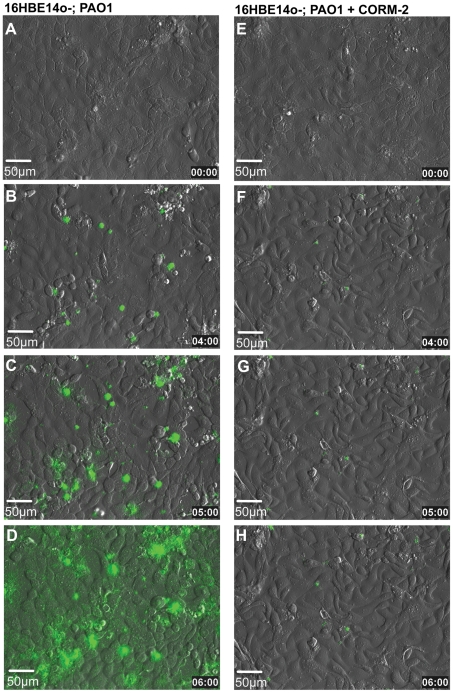
CORM-2 attenuates *P. aeruginosa* colonization of respiratory epithelium. Time –lapse microscopy of bronchial epithelial cells co-cultured with PAO1 (1∶3 ratio) show that the addition of 50 µM of CORM-2 reduced microcolony formation. The time (hours) when the images were acquired is indicated at the bottom of each panel. Movies are available as supplementary online data (Movies S1 and S2). Scale bars = 50 µm.

We observed that CORM-2 prevents the development of mature biofilms on plastic and glass. We next decided to examine the effect of CORM-2 on bacterial colonization of human bronchial epithelial cells. PAO1-GFP bacteria attached to 16HBE14o- cells were treated with 50 µM of CORM-2 or DMSO and microcolony formation was followed for 6 h with time-lapse microscopy. The addition of 50 µM CORM-2 inhibited bacterial growth resulting in reduced microcolony formation and less damage to the epithelial cells when compared with DMSO addition (Figure 4 A–H and Movies S1 and S2) or iCORM (data not shown). Epithelial cell integrity was continuously assessed by differential interference contrast.

**Figure 5:**
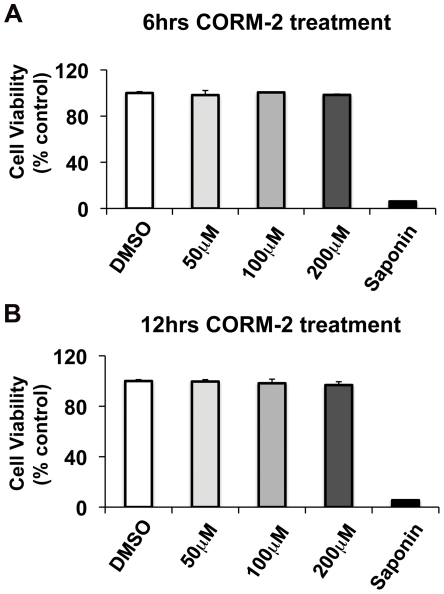
CORM-2 does not affect airway epithelial cell viability. The MTT viability assay shows that 50–200 µM of CORM2 is not toxic to airway epithelial cells after 6 hours (A) or 12 hours (B) treatment. As a control for cell death, 16HBE14o- cells were treated with saponin for 10 minutes before proceeding with the MTT staining. CORM-2 viability is expressed as percentage of non-treated control cells (DMSO).

Previous work has shown that CO-RM molecules are not toxic to mammalian cells [16], [23] or when used intravenously in animal models [7]. We tested whether CORM-2 has a cytotoxic effect on airway epithelial cells. 16HBE14o- cells were treated with 50 µM, 100 µM and 200 µM of CORM-2 for 6 h or 12 h. Control cells were treated with vehicle alone (DMSO). CORM-2 was not associated with any cell toxicity at the doses tested either after 6 h or 12 h exposure (Figure 5 A, B). These data reveal that at bactericidal concentrations (50 µM– 200 µM) CORM-2 is effective in disrupting *P. aeruginosa* surface colonization of biotic surfaces at doses that are not toxic for human bronchial epithelial cells.

### CORM-2- and tobramycin-mediated antimicrobial activities are additive

**Figure 6:**
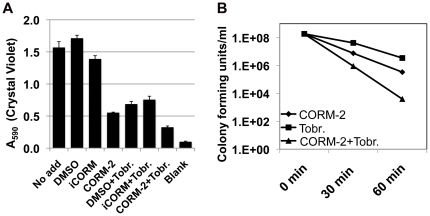
CORM-2 and tobramycin have additive effects on PAO1 biofilm formation. A) CORM-2 (100 µM) is as effective as tobramycin (1 mg/ml) in decreasing the biomass of the bacterial biofilm after 6 hours treatment, as measured by CV staining and the combination is better than either alone. B) The combination of CORM-2 (100 µM) and tobramycin (Tobr.) results in increased PAO1 killing within the biofilm than either drug alone.

Given the difficulty in treating bacterial biofilms, we next sought to determine if CORM-2 would work as an adjuvant therapeutic agent with currently available antimicrobials. Tobramycin is an aminoglycoside antibiotic commonly given by nebulizer to patients with chronic pulmonary *P. aeruginosa* infection. One hundred µM CORM-2 is as effective as 1 mg/mL tobramycin at decreasing biofilm biomass as measured by CV staining (Figure 6A). The addition of CORM-2 (100 µM) and tobramycin (1 mg/ml) in combination for 6 h resulted in decreased CV staining of PAO1 biofilms compared with either no drug or either drug alone with no recovery of viable bacteria (Figure 6A and data not shown)(p<0.05 ANOVA, Dunnett's post-test analysis). To determine whether there was a difference in the recovery of viable bacteria comparing single and combination treatments, we lowered the dose of tobramycin to 100 µg/ml. Exposure to 100 µg/ml tobramycin resulted in a one-log drop in bacterial viability by 60 min, while exposure to CORM-2 led to a two-log drop at 60 min (Figure 6B). Exposure to both drugs resulted in an additive effect with a four-log drop in recovered viable bacteria at 60 min (Figure 6B), as assessed by colony forming units assay. Bacterial recoveries from all treatments except the 30-minute tobramycin group were significantly different than the number of bacteria recovered at t_0_ (ANOVA<0.001, Dunnett's post-test analysis).

### Antioxidants protect PAO1 biofilms from CORM-2 inhibition

**Figure 7:**
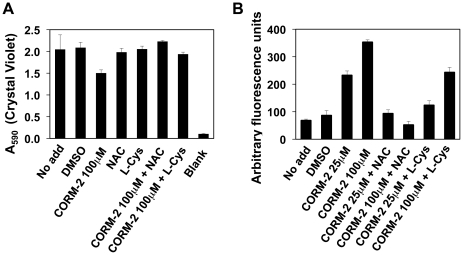
Anti-oxidants protect *P. aeruginosa* biofilms from CORM-2 inhibition. A) The addition of NAC (1 mM) or L-cysteine (100 µM) to CORM-2 (100 µM) treated *P. aeruginosa* biofilms (6 hours) restores CV staining to levels comparable to untreated biofilms. B) Treatment with CORM-2 (25 µM and 100 µM) induces bacterial ROS formation; NAC (1 mM) prevents ROS production while the addition of L-cysteine (100 µM) did alter only partially ROS levels.

Previous work on planktonic *P. aeruginosa* demonstrated that multiple reducing agents (NAC, L-cysteine, and reduced glutathione) prevent *P. aeruginosa* killing by CO-RMs [18]. Our initial studies of planktonic *P. aeruginosa* and CORM-2 with NAC or L-cysteine confirmed this protection (data not shown). We next added NAC or L-cysteine in combination with CORM-2 during biofilm formation in M9 medium to determine whether the treated biofilms were protected against CORM-2 induced cell death. Indeed, CV staining of PAO1 biofilms formed in 96-well plates exposed to either NAC or L-cysteine in the presence of CORM-2 was similar to untreated biofilm formation (Figure 6A). CORM-2 treatment alone showed a significant decrease in CV staining compared with controls (no drug or DMSO) (Figure 7A) (p<0.01 ANOVA, Dunnett's post-test analysis).

Published reports have suggested multiple mechanisms of the bactericidal action of CO-RMs including ROS-mediated activity for CORM-2 [18], [20], [21]. ROS production by PAO1 biofilms increased after CORM-2 exposure >10 µM as assessed by DCF fluorescence intensity compared with untreated biofilms (Figure 7B, ANOVA p<0.001, Dunnett's post test analysis). Interestingly, while the addition of NAC to PAO1 biofilms prevented ROS production, the addition of L-cysteine only reduced ROS production to levels seen when 25 µM CORM-2 alone is added (Figure 7B). At this concentration, CORM-2 was sufficient to reduce CV staining (Figure 2A). L-cysteine with 100 µM CORM-2 resulted in no statistical significant decrease in biofilm formation compared with control samples despite similar levels of ROS production (Figure 7A) (N.S. by ANOVA). Therefore, our data support the previous observations that ROS production is not the major mechanism by which CORM-2 disrupts *P. aeruginosa* growth in M9 medium with glucose and an alternative mechanism such as binding of CO to *P. aeruginosa* respiratory chain is more likely [22].

### Rich medium protects against CORM-2-dependent PAO1 death

**Figure 8:**
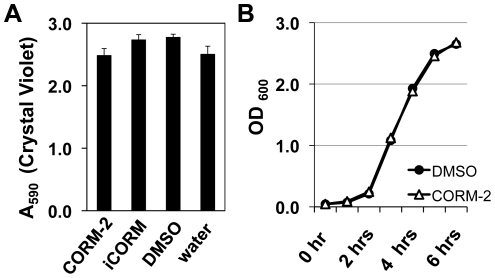
Rich medium protect *P. aeruginosa* biofilms against CORM-2. A) Biofilm formation by *P. aeruginosa* treated with CORM-2 (100 µM) for 6 hours is similar to untreated controls when grown in LB. B) The addition of CORM-2 (100 µM) to *P. aeruginosa* growing in liquid LB medium does not result in cell death.

We next examined biofilm formation in rich medium, where an increase in biofilm formation has been observed for *E. coli* after CORM-2 addition [21]. In contrast to *E. coli*, PAO1 biofilm formation in LB does not change significantly after addition of CORM-2 (Figure 8A). Planktonic PAO1 grown in shaking LB were also protected from CORM-2-dependent cell death (Figure 8B). ROS production by PAO1 biofilms grown in LB was slightly increased after exposure to 100 µM CORM-2 but not to the same extent as biofilms grown in minimal media (data not shown). The nutritional conditions and composition of the growth medium are therefore critically important in determining the activity of CORM-2 during both liquid and surface associated growth and this likely has implications [20] for *in vivo* efficacy.

### CORM-2 has differential effects on biofilm formation and planktonic growth of clinical isolates and PA14

**Figure 9:**
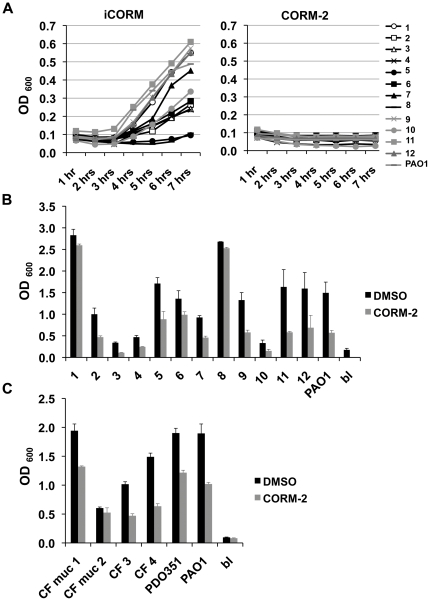
The effect of CORM-2 on growth and biofilm formation of clinical, respiratory *P. aeruginosa* isolates. A) The addition of 100 µM CORM-2 to M9 liquid medium with glucose (left) prevents the growth of all clinical isolates compared with addition of DMSO alone (right). B) 6 hours exposure to 100 µM CORM-2 reduces the CV staining of 10/12 biofilms formed by respiratory isolates after overnight growth in plastic wells. C) 100 µM CORM-2 reduces the CV staining of 3 out of 4 biofilms formed by respiratory isolates from CF patients independently of the mucoid or non-mucoid after overnight growth in plastic wells. A similar effect was observed in mucoid-PAO1 laboratory strain PDO351 (mucA:aac+;alg+).

CORM-2 prevents PAO1 planktonic growth and biofilm maturation. Given the genetic and phenotypic diversity of *P. aeruginosa* [24] we next tested the effect of CORM-2 on liquid growth and the biofilm formation of a number of previously characterized clinical isolates from respiratory specimens of non-CF patients [25]. Two of twelve respiratory isolates grew poorly in liquid M9 minimal medium (isolates #5 and #8) (Figure 9A, left panel). Of the remaining ten, growth of all strains was inhibited by the presence of 100 µM CORM-2 (Figure 9, right panel). Ten of the twelve (83%) strains grown overnight in plastic wells displayed statistically significant reduced CV staining of the biofilm after 6 h incubation with 100 µM CORM-2 compared with no treatment (Figure 9B) (p<0.05, paired t-tests). Three isolates (#3, #4, #10) formed poor biofilms both in the absence of CORM-2 and when CORM-2 was added. For these three isolates, the CV staining of the CORM-2 treated biofilms was similar to the un-inoculated blank. Two strains unaffected by CORM-2 (#1, #8) formed robust biofilms compared with PAO1 during standing growth in M9 with glucose (Figure 9B). These data suggest that while the majority of respiratory isolates are susceptible to CORM-2 treatment, isolates with robust biofilm formation are less likely to be affected by CORM-2 treatment. Interestingly, isolate #8 had poor planktonic growth but robust biofilm formation in M9 with glucose. These data highlight the role of biofilm formation in the process of *P. aeruginosa* strain adaptation to unfavorable metabolic conditions.

The above strains were from patients without CF and more likely represent environmental strains and not strains from patients with chronic infection. Chronic pulmonary *P. aeruginosa* infection often results in the selection of alginate producing mucoid strains [26]. We next examined whether CORM-2 alters the biofilm formation of isolates recovered from CF patients. We included both mucoid and non-mucoid clinical isolates from CF patients along with PDO351(mucA:aac+;alg+), a mucoid strain that is otherwise isogenic to PAO1 [27] (Figure 9C). Similar to the results for the non-CF *P. aeruginosa* isolates we found that CORM-2 reduced biofilm formation in some strains but not all (Figure 9C) (p<0.012, paired t-test). The efficacy of CORM-2 did not correlate with the mucoid status of the isolate; for example, CORM-2 had the same effects on mucoid PDO351 compared with non-mucoid wild type PAO1 while the biofilm formation of other mucoid clinical strains were unaffected by the addition of CORM-2 (Figure 9C). Given that biofilms may form under anaerobic conditions during chronic pulmonary infection, we assayed anaerobic planktonic growth in M9 glucose or LB medium using 40 mM potassium nitrate as an electron acceptor. Addition of 100 µM CORM-2 inhibited growth of PAO1 in M9 medium but not LB (as observed for aerobic conditions), demonstrating CORM-2 activity at different oxygen tensions (Figure S2).

**Figure 10:**
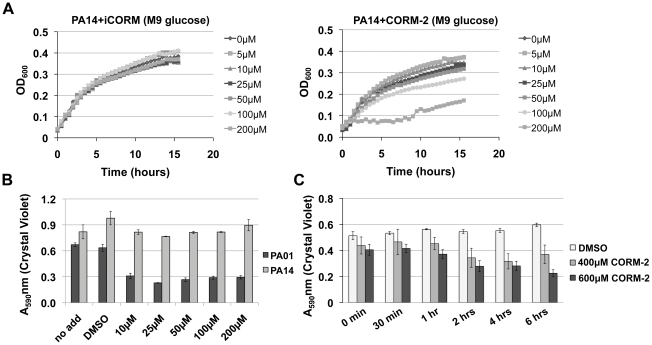
High doses of CORM-2 are required to reduce growth and biofilm formation of PA14. A) CORM-2 doses >100 µM (right panel) but not iCORM (left panel) prevents PA14 growth in liquid M9 medium with glucose. B) As measured by CV staining, PA14 biofilms are resistant to the low doses of CORM2 treatment (10–200 µM) that reduce PAO1 biofilm formation. C) Higher doses of CORM-2 (400 µM and 600 µM) decreases CV staining of both PA14 and PAO1 biofilm formation. Each condition was tested in triplicate.

Given the heterogeneity of the effects of CORM-2 on clinical isolates we next tested the effect of CORM-2 on another commonly used laboratory strain, PA14 (Figure 10). Similarly to clinical isolates #5 and #8, PA14 displayed poor planktonic growth in M9 glucose medium when compared with PAO1 (data not shown). PA14 was relatively resistant to CORM-2 and to prevent planktonic PA14 growth 200 µM CORM-2 was required; iCORM did not have any effect on PA14 growth at any concentration (Figure 10A). Interestingly, while planktonic PA14 growth was poor in M9, PA14 formed strong biofilms after overnight growth in M9 similarly to what observed for PAO1 (Figure 10B). Thus, PA14 displays adaptation to unfavorable metabolic conditions as also observed for clinical isolates #5 and #8. PA14 biofilms were insensitive to low doses of CORM-2 (10–200 µM) (as also observed for clinical isolated #8), and required much higher doses of CORM-2 (400–600 µM) to inhibit biofilm formation compared with PAO1 (Figure 10C). These data suggest that intrinsic differences in *P. aeruginosa* strains affect their susceptibility to CORM-2. Since we observed different growth rates between PAO1 and PA14 stains in minimal M9 medium supplemented with glucose, we hypothesized that the susceptibility to CORM-2 is related to the strain-related capability of using different carbon sources for energy. Our finding that CORM-2 becomes ineffective when either PAO1 or PA14 is grown in rich LB media (Figure 8 and data not shown) also supports this hypothesis.

## Discussion

In patients with CF, there is a chronic hyper-inflammatory state within the infected lung that fails to eradicate colonization with pathogens such as *S. aureus* and *P. aeruginosa* leading to clinical symptoms and frequent hospitalizations [5]. Chronic colonization is initiated with surface attachment followed by microcolony formation, the precursors to the mature, antibiotic-resistant biofilm [4], [5]. Since mature biofilms are difficult to eradicate, an alternative approach to therapy is to generate novel antimicrobial agents to prevent these early stages of biofilm formation enhancing the efficacy of the immune response. CO is an appealing therapeutic agent because it has the potential to reduce both host pulmonary inflammation and bacterial viability [15]. CO-RMs were developed to improve and maximize the effects of CO delivery to tissues and CORM-2, used in this study, can efficiently release controlled amount of CO with the same cytoprotective activities that are described by exogenous administration of CO gas without any cytotoxic effect on mammalian cells [16].

We demonstrate that, in addition to killing planktonic *P. aeruginosa*, CORM-2 also prevents microcolony formation on human bronchial epithelial cells while maintaining the integrity of the epithelium (Figure 4). Importantly, CORM-2 concentrations required for *P. aeruginosa* killing were not toxic to human airway epithelial cells even after a 12 hours exposure (Figure 5). We cannot exclude that CORM-2 prevented epithelial cells disruption not only by impairing bactericidal growth, but also by enhancing the antimicrobial activity of mammalian cells. This is an area of active investigation.

CORM-2 also attenuates *P. aeruginosa* biofilm formation on abiotic surfaces confirming the antimicrobial effect beyond that previously shown for planktonic bacteria [17], [18] (Figure 2). Together, these data suggest CORM-2 can inhibit biofilm formation regardless of the surface used for bacterial attachment. Despite the genetic and phenotypic diversity of *P. aeruginosa*, the majority of clinical isolates from non-CF patients tested had decreased biofilm formation after exposure to CORM-2 (Figure 7). Importantly, pulmonary infections in CF patients are usually initiated by environmental *P. aeruginosa* strains that often cause other types of human infection. The two clinical isolates resistant to CORM-2 formed robust biofilms rapidly when compared with the other isolates, suggesting that the earlier CORM-2 is applied the more effective it is likely to be. CORM-2 also attenuated the biofilm formation of a subset of isolates from CF patients, including both non-mucoid and mucoid strains. While mucoid and non-mucoid strains have multiple phenotypic and genetic differences, mucoidy did not predict failure of CORM-2 treatment [28]. Interestingly, the commonly used laboratory strain PA14 showed increased resistance to CORM-2 exposure. We hypothesize this is due to its slow growth rate in the presence of glucose and its ability to adapt to unfavorable metabolic conditions. Additional studies are required to determine whether metabolic differences between PA14 and PAO1 are responsible for their different susceptibility to CORM-2 treatment.

The combination of CORM-2 with tobramycin, an antibiotic currently used in therapy for *P. aeruginosa* pulmonary infection in CF patients, was more effective at killing bacteria and reducing biomass than either drug alone (Figure 6). To our knowledge this is the first example of an added benefit when combining CORM-2 with another antimicrobial agent suggesting CORM-2 may have a role as an adjunct therapy with currently available antimicrobials. This may be particularly helpful during chronic infections where subpopulations of antibiotic resistant bacteria persist after treatment.

Exposure of *E. coli* to CORM-2 results in increased ROS production, DNA damage and intracellular iron levels of iron [20], suggesting that ROS generation is the primary mechanism of cell death [20]. ROS production does not appear to be the mechanism of CORM-2 dependent PAO1 cell death in liquid growth [22]. Interestingly, while ROS production is increased from *P. aeruginosa* biofilms after CORM-2 exposure, the addition of L-cysteine only partially inhibits ROS production but completely protects *P. aeruginosa* biofilm formation from the effects of CORM-2 (Figure 7). This suggests that ROS do not cause cell death in CORM-2 treated biofilms. The mechanism of protection provided by reducing agents to *P. aeruginosa* observed by us and others remains unknown [20]. Microarray analysis of *E. coli* exposed to CO-RMs also demonstrate a global effect with transcriptional changes in a wide variety of metabolic pathways and transcriptional regulators, including those involved in carbohydrate production, energy metabolism, and stress response [19], [21]. Whether any of the above transcriptional changes occur in *P. aeruginosa* and contribute to CORM-dependent cell death is unclear.

Exposure to CO-RMs has been shown to result in CO binding to components of the respiratory chain in both *E. coli* and *P. aeruginosa* [18], [19]. The *P. aeruginosa* respiratory chain is complex and involves a large number of respiratory oxidases [29]. At least two cytochrome oxidases interact with CORM-3 as assayed via dual wave spectrophotometry [18]. Exposure of planktonic cultures to CORM-3 results in a temporal decrease in oxygen consumption consistent with the hypothesis that interference of CO with the respiratory chain kills *P. aeruginosa* [18]. CORM-2 inhibits *E.coli*, *Camplyobacter jejuni* and *P. aeruginosa* PAO1 also under anaerobic conditions [19], [30], suggesting that oxygen is not required as the terminal electron acceptor for CORM-2 activity.

In LB-rich medium *P. aeruginosa* planktonic growth and biofilm formation are unaffected by CORM-2. This is in contrast to *E. coli* where CO-RMs increase both oxidative stress and biofilm formation [21]. The reason CORM-2 is not effective during growth in rich medium is unknown. One hypothesis currently being tested is that the active energy pathways used by *P. aeruginosa* determine its susceptibility to CORM-2 and are dependent on the nutritional environment and bacterial growth rate. Given the genetic and phenotypic variability of *P. aeruginosa*, these observations are of particular importance since intrinsic differences in metabolism amongst *P. aeruginosa* strains may limit the effectiveness of therapies that target energy production.

In conclusion, this report demonstrates that, in addition to infections that likely involve planktonic bacteria such as bacteremia, CO-RMs are a potential therapeutic option for *P. aeruginosa* surface-associated infections with a biofilm component such as pulmonary and perhaps burn infections [18]. Since CORM-2 demonstrated activity against mucoid strains and under anaerobic conditions it has the potential to have an effect on chronic *P. aeruginosa* pulmonary infections as observed in patients with long-standing lung disease such as CF. However, as several clinical isolates and PA14 were resistant to CORM-2 dependent killing, this compound will not work for all infections and the emergence of resistant strains is possible. We hypothesize that the nutrients available and the energy pathways used by *P. aeruginosa* in the host during infection will be critical in determine the potential for CO-RMs as *in vivo* therapeutics. This is an important area for additional studies.

Importantly, a single dose of CORM-2 achieved significant bacterial killing for most isolates. If CORM-2 were dosed more frequently, we hypothesize it will result in additional bacterial death within the biofilm. Current efforts are underway to improve CORM-2-dependent biofilm disruption and better understand the mechanisms of both CORM-2 action within *P. aeruginosa* and CORM-2 resistance in non-susceptible strains or in different metabolic conditions.

## Methods

### Drugs

CORM-2, which contains a ruthenium metal surrounded by six carbonyl (CO) groups, was dissolved in DMSO (stock solution 10 mM) (Sigma-Aldrich, St Louis MO). As a negative control for CORM-2, an inactive compound, here referred to as iCORM, was used (stock solution 10 mM). This compound [Ru(DMSO)_4_Cl_2_) consists of a ruthenium metal where the CO groups have been replaced by DMSO [11]. Both drugs were diluted to final concentrations of 10–600 µM, depending on the experiment. Stocks were prepared fresh for each experiment. Tobramycin (stock solution 100 mg/ml) (MP Biomedicals, Solon OH) was used at final concentrations ranging from 100 µg to 1 mg/ml, depending on the experiment. N-acetyl cysteine (NAC) was added at a final concentration of 1 mM and L-cysteine was used at 100 µM [18].

### Strains and Growth Conditions

PAO1 is the reference laboratory strain used in all experiments except where described. In some experiments, the mucoid PDO351(mucA:aac+;alg+) strain, derived from PAO1, was used [27]. The effect of CORM-2 on clinical isolates was determined using previously characterized *P. aeruginosa* respiratory isolates from patients without CF [25] and isolates from patients with CF. M9 salts growth medium was supplemented with 0.4% glucose. Vogel Bonner Medium (VBM) agar plates selective for *P. aeruginosa* were made as described [25]. The expression of the yellow fluorescent protein (YFP) from the plasmid pMQ72 in PAO1 was induced by supplementing the media with arabinose (0.02%) [31]. PAO1 expressing green fluorescent protein (GFP) from the high copy plasmid pUCPSK was grown overnight in the presence of carbenicillin (200 µg/ml). The PAO1-GFP bacteria were washed and resuspended in M9 medium without carbenicillin prior to incubation with respiratory epithelial cells. For growth experiments, overnight cultures of PAO1 were diluted into fresh medium to an optical density (OD) at 600 nm of 0.05, the CORM-2, iCORM, or DMSO added, and the OD followed over time. At specified intervals, aliquots were removed from the cultures, diluted to VBM agar plates, and incubated overnight at 37°C to determine the number of viable bacteria. In some growth experiments, another laboratory strain, PA14, was used. PA14 was also diluted to an OD (600 nm) of 0.05 from overnight cultures grown aerobically at 37°C in Luria-Bertani broth (EMD) or M9 salts growth medium was supplemented with 0.4% glucose. The CORM-2, iCORM or DMSO treatment was added and the OD at 600 nm was followed over time using a Biotek Synergy 4 spectrophotometer.

The effect of CORM-2 on PAO1 growth in anaerobic conditions was also tested. Luria-Bertani broth (EMD) and M9 medium with 0.4% glucose as the carbon source both supplemented with 40 mM KNO_3_ were autoclaved and moved into a Coy anaerobic chamber (80%/15%/5% N_2_/CO_2_/H_2_ atmosphere) 16 hours before inoculation. Pre-cultures were grown aerobically overnight and moved into the anaerobic chamber for use as inocula. Anaerobic media were dispensed 5 mL per 18×150 mm anaerobic Balch tube (Bellco) and inoculated to a final OD (600 nm) of 0.05. Cultures were stoppered and crimp-sealed in the chamber, then incubated at 37°C with shaking at 250 rpm. OD at 600 nm was followed using a Thermo Spectronic 20D+ spectrophotometer.

### Ethics Statement

Clinical *P. aeruginosa* isolates were taken from de-identified frozen stocks conserved at −80°C as part of a previous study approved by the Human Investigations Committee at Yale. These bacteria were recovered from pediatric and adult patients hospitalized at Yale-New Haven Hospital and identified by the clinical microbiology laboratory, as described in previously in detail [25]. All patients or their legal guardians provided informed consent.

### Static biofilm formation and analysis

PAO1 static biofilms were formed in either 24-well plastic dishes (Corning, Lowell MA) or 96-well plastic dishes (Grenier bio-one, Germany). PA14 static biofilms were formed in 96-well plastic dishes (Grenier bio-one, Germany); the bacteria were inoculated from an overnight shaking culture to an OD (600 nm) of 0.05 and then incubated as a static (non-shaking) culture from 18 to 24 hours at 37°C [32]. Under these conditions, a biofilm forms around the edge of the miniscus against the wall of the well. CORM-2, iCORM, DMSO, and/or tobramycin were added directly without removing planktonic cells after biofilm formation. In a different set of experiments, the planktonic cells were removed and the remaining biofilm was gently washed before adding fresh medium with CORM-2 or controls (DMSO or iCORM). At selected times, the biofilm was washed in water, stained with 0.05% crystal violet (CV) and absorption at 595 nm was recorded [32]. For a single experiment, the drug additions were tested in triplicate and all experiments were repeated at least three times.

To recover viable bacteria from mature biofilms, the planktonic culture was removed after overnight growth, the biofilms were gently washed, and fresh medium with drug was added for 30 min to 6 hours. Following this incubation, the biofilms were washed 4 times with fresh medium, then sonicated to release bacteria [32]. The medium containing the released bacteria was serially diluted and plated on VBM agar. In all experiments, 3 wells on each plate were initially washed and sonicated to determine the viable cell counts at time point t_0_.

### Analysis of biofilm formation in real time

For real-time imaging of the effects of CORM-2 on biofilm formation, PAO1-YFP was grown overnight in glass bottom poly-lysine coated dishes (MatTek, Ashland MA) [33] and then placed in a VivaView incubator fluorescent microscopy system (Olympus, Center Valley PA), and equilibrated at 37°C. CORM-2 (50 µM), iCORM (50 µM), tobramycin (100 µg/ml) or DMSO was added and images were collected every 15 minutes for 8 hours. The images from three fields for each sample were collected and analyzed with VivaView software.

### Microcolony formation on respiratory epithelial cells

The static bacteria/bronchial epithelial cell co-culture system was established as described [33]. 16HBE14o- bronchial epithelial cells (a kind gift of Dr. Dieter Gruenert) were maintained in Minimum Essential Medium supplemented with 10% fetal bovine serum, L-glutamine and penicillin/streptomycin. For time-lapse microscopy experiments, 1×10^6^ cells were plated in collagen/fibronectin coated glass bottom 3 cm dishes (MatTek, Ashland MA) and cultured for 5–7 days, allowing for tight junction formation. Subsequently the epithelial cells were inoculated with *P. aeruginosa*-GFP at a ratio of 1∶3 in media without antibiotics. After 40 minutes in culture, unattached planktonic *P. aeruginosa* were removed by rinsing and replaced with fresh M9 medium supplemented with glucose, L-glutamine and 0.04% L-arginine. CORM-2 (50 µM), iCORM (50 µM) or DMSO was added and images were collected by time-lapse microscopy (VivaView, Olympus) every 10 minutes for 6 hours (objective 20×). The epithelium integrity was assessed by differential interference contrast and remained intact for 7–8 hours of culture in the absence of bacteria. The images from three fields for each sample were collected and analyzed with VivaView software.

### Cell Viability Assay

16HBE14o- bronchial epithelial cells were seeded in 96-wells plate (5×10^4^ cells/well). After 5 days of culture in Minimum Essential Medium supplemented with 10% fetal bovine serum, L-glutamine and penicillin/streptomycin, cells were treated with different doses of CORM-2 (50 µM, 100 µM and 200 µM) or with vehicle alone (DMSO) for 6 hours or 12 hours. The cell viability was then assessed using the MTT (3-(4,5-Dimethylthiazol-2-yl)-2,5-Diphenyltetrazolium Bromide) colorimetric assay, which measures cellular metabolic activity. As control for cell death, 16HBE14o- cells were treated with saponin for 10 minutes before proceeding with the MTT staining. The MTT staining and the colorimetric detection (Cell Biolabs, INC) were performed following the manufacturer's instructions. Each condition was performed in triplicate.

### Measurement of ROS production in bacteria

To determine ROS production, static biofilms were grown in 96-well plastic dishes (Corning, Lowell MA) as described above [32]. Biofilms were washed gently and CORM-2 (25 µM or 100 µM) or DMSO in the absence or presence of NAC (1 mM) or L-cysteine (100 µM) were added to the biofilm for 2 hours at 37°C. Biofilms were then washed in Phosphate Buffered Saline (PBS) and loaded with 10 µM 5-(and-6)-chloromethyl-2′,7′-dichlorodihydrofluorescein diacetate, acetyl ester (CM-H2DCFDA; Molecular Probes) for 30 min at 37°C. This was followed by two washes in PBS. In the presence of intracellular ROS and esterases, CM-H2DCFDA is oxidized and deacetylated yielding the fluorescent molecule 2′,7′-dichlorodihydrofluorescein diacetate (DCF). Fluorescence intensity was assessed using a fluorescence plate reader (arbitrary units).

### Statistical analysis

Data was compiled in Excel and transferred to Minitab for statistical analysis. For data derived from biofilm analysis using the CV method, for analysis of ROS production and for the analysis of cell viability statistical comparison of paired means was done with a paired t-test. When required, multiple comparisons of means were done using a one-way ANOVA. Post-test analysis determining confidence intervals for individual means was done with Dunnett's analysis comparing treated samples to the control.

## Supporting Information

Figure S1 **CORM-2 attenuates PAO1 biofilm formation.** (A) Mean fluorescence intensity (MFI) of YFP-*P. aeruginosa* grown for 16 hours in a glass bottom dish and then visualized at selected locations for 12 additional hours using VivaView. MFI over time was normalized to the MFI at time 0 h. CTR = no addition control; iCORM (50 µM); CORM-2 (50 µM). (B) P values for each group comparison are shown (t-test).
(PDF)

Figure S2 **CORM-2 inhibits anaerobic growth of PAO1 in M9 glucose medium.** PAO1 was grown anaerobically in M9 glucose or LB medium in the presence of 40 mM nitrate. Addition of CORM-2 (100 µM) inhibited planktonic growth of PAO1 in M9 but not in LB.
(PDF)

Movie S1 **CORM-2 attenuates PAO1 microcolony formation on human bronchial epithelial cells.** Time–lapse microscopy of bronchial epithelial cells co-cultured with PAO1 (1∶3 ratio) in presence of vehicle control (DMSO).
(MP4)

Movie S2 **CORM-2 attenuates PAO1 microcolony formation on human bronchial epithelial cells.** Time–lapse microscopy of bronchial epithelial cells co-cultured with PAO1 (1∶3 ratio) in presence of CORM-2 (50 µM). CORM-2 reduced microcolony formation.
(MP4)
