# Supplementary material for: The Carbon Monoxide Releasing Molecule CORM-2 Attenuates Pseudomonas aeruginosa Biofilm Formation
Source: PLoS One. 2012 Apr 26;7(4):e35499. doi: 10.1371/journal.pone.0035499 (PMC3338523; doi:10.1371/journal.pone.0035499)
Supplement: Figure S1 — CORM-2 attenuates PAO1 biofilm formation. (A) Mean fluorescence intensity (MFI) of YFP-P. aeruginosa grown for 16 hours in a glass bottom dish and then visualized at selected locations for 12 additional hours using VivaView. MFI over time was normalized to the MFI at time 0 h. CTR = no addition control; iCORM (50 µM); CORM-2 (50 µM). (B) P values for each group comparison are shown (t-test). (PDF) [file pone.0035499.s001.pdf]

A

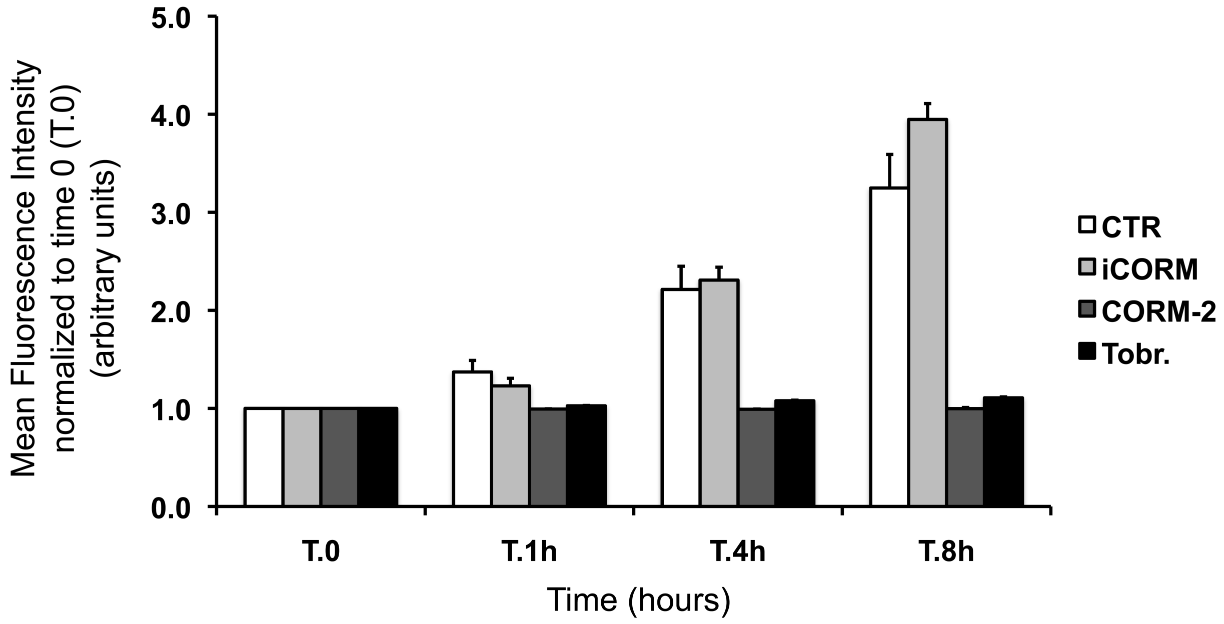

B

**T-TEST**	T.1h	T.4h	T.8h	
CTR vs iCORM	0.07795	0.28702	0.01649	p values
CTR vs CORM-2	0.00257	0.00043	0.00017	p values
CTR vs Tobramycin	0.00356	0.00057	0.00021	p values
CORM-2 vs Tobramycin	0.00006	0.00002	0.00011	p values

Figure S1. CORM-2 attenuates PAO1 biofilm formation. (A) Mean fluorescence intensity (MFI) of YFP-*P.aeruginosa* grown for 16 hrs in a glass bottom dish and then visualized at selected locations for 12 additional hours using VivaView. MFI over time was normalized to the MFI at time 0h. CTR = no addition control; iCORM (50µM); CORM-2 (50µM). **(B)** P values for each group comparison are shown (t-test).
