# Supplementary material for: The Carbon Monoxide Releasing Molecule CORM-2 Attenuates Pseudomonas aeruginosa Biofilm Formation
Source: PLoS One. 2012 Apr 26;7(4):e35499. doi: 10.1371/journal.pone.0035499 (PMC3338523; doi:10.1371/journal.pone.0035499)
Supplement: Figure S2 — CORM-2 inhibits anaerobic growth of PAO1 in M9 glucose medium. PAO1 was grown anaerobically in M9 glucose or LB medium in the presence of 40 mM nitrate. Addition of CORM-2 (100 µM) inhibited planktonic growth of PAO1 in M9 but not in LB. (PDF) [file pone.0035499.s002.pdf]

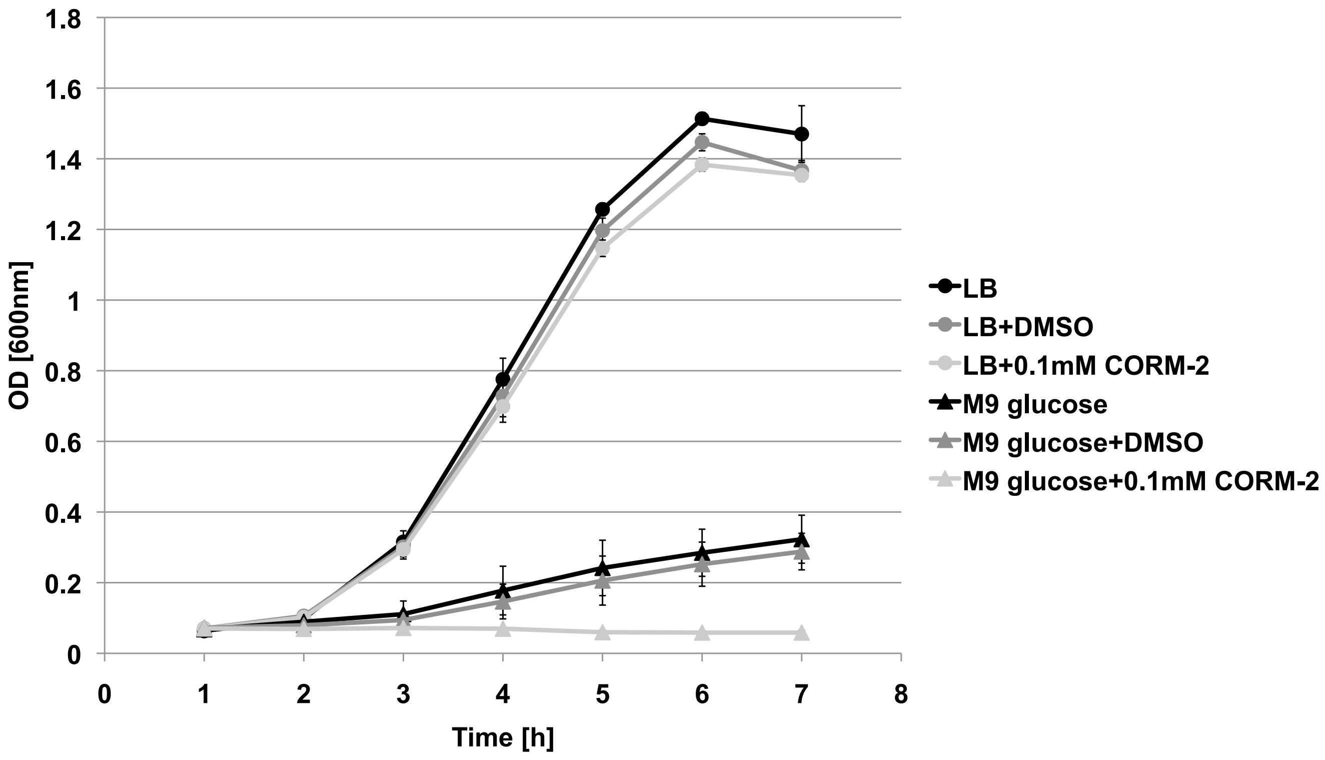

Figure S2. CORM-2 inhibits anaerobic growth of PAO1 in M9 glucose medium. PAO1 was grown anaerobically in M9 glucose or LB medium in the presence of 40 mM nitrate. Addition of CORM-2 (100µM) inhibited planktonic growth of PAO1 in M9 but not in LB.
